# Parental Psychological Profiles in Autism and Other Developmental Contexts: A Latent Profile Analysis Informing Coordinated Family-Centered Care

**DOI:** 10.3390/children13060740

**Published:** 2026-05-26

**Authors:** Margarita Bakracheva, Elena Ivanova, Kaloyan Damyanov, Rositsa Racheva, Milen Zamfirov

**Affiliations:** 1 Faculty of Educational Studies and the Arts, Sofia University “St. Kliment Ohridski”, 15 Tzar Osvoboditel Str., 1504 Sofia, Bulgaria; kdamjanov@uni-sofia.bg (K.D.); m.zamfirov@fppse.uni-sofia.bg (M.Z.); 2 University Hospital “Alexandrovska”, 1 “St Georgi Sofiiski” Str., 1431 Sofia, Bulgaria; helen_avian@abv.bg; 3 Institute of Population and Human Studies, Bulgarian Academy of Sciences, 6 Acad. Georgi Bonchev Str., 1113 Sofia, Bulgaria; rositsa.racheva@abv.bg

**Keywords:** parenting, developmental disorders, psychiatric diagnosis, family functioning, family-centred care

## Abstract

**Highlights:**

**What are the main findings?**

- Three distinct parental profiles were identified—Low well-being, high co-dependency and low resource functioning; high well-being, low co-dependency and high resource functioning; and Moderate well-being, moderate co-dependency and moderately resourceful functioning—revealing substantial heterogeneity across clinical and typical developmental contexts.
- Parents of children with psychiatric disorders were significantly more likely to belong to the most vulnerable Low well-being, high co-dependency and low resource functioning profile, highlighting specific psychological strain.

**What are the implications of the main findings?**

- Parents of typically developing children more often belong to the Moderate well-being, moderate co-dependency and moderately resourceful functioning profile, indicating that parents in general may need support to build resources, regardless of diagnostic status.
- The findings reveal the key significance of family-centered care, particularly for families raising children with ASD and psychiatric diagnoses. The identified profiles provide a foundation for future research across all family contexts within a family-centered framework, supporting the identification of vulnerable parents and guiding targeted family support.

**Abstract:**

**Background:** Autism spectrum disorder (ASD) or psychiatric conditions, special education needs and addictions exert substantial demands on families, yet parental psychological functioning remains insufficiently integrated into prevention and family-based support. An in-depth understanding of parental experiences constitutes a cornerstone of family-centered care, given that parental well-being and resourceful functioning exert a direct and enduring influence on both intervention effectiveness and the broader trajectory of child development. This study aimed at identifying latent parental profiles across clinical and typical developmental contexts. **Methods:** A total of 281 parents of children with psychiatric diagnoses (primarily ASD), special educational needs, addiction, and typical development were assessed for positive and negative functioning. Latent Profile Analysis (LPA) identified psychological subgroups, followed by Welch’s ANOVA to determine discriminating variables and multinomial logistic regression to examine sociodemographic and contextual predictors. **Results:** A three-profile solution emerged: Low well-being, high co-dependency and low resource functioning (50%); High well-being, low co-dependency and high resource functioning (26%); and Moderate well-being, moderate co-dependency and moderately resourceful functioning (24%). Parents of children with psychiatric diagnoses were significantly less likely to belong to the High well-being, low co-dependency and high resource functioning profile, underscoring the heightened psychological vulnerability characteristic of this group. Parents of typically developing children tend to belong to Moderate well-being, moderate co-dependency and moderately resourceful functioning. **Conclusions:** Parental psychological functioning exhibits heterogeneity across developmental contexts. Although parents of children with psychiatric diagnoses revealed the highest vulnerability, the profiles also revealed substantial psychological strain among parents of typically developing children. These findings highlight the need to shift from child-focused to family-centered prevention and support.

## 1. Introduction

The child’s clinical diagnosis reshapes the entire family system, and although clinical practice traditionally prioritizes child-centered outcomes, parental psychological health remains a fundamental determinant of long-term intervention success. Recent evidence suggests that parental self-regulation directly influences the child’s developing sense of security [1]. While modern parenting challenges—such as technological immersion and societal disruptions—undermine well-being across typical developmental contexts [2,3], these stressors are markedly amplified for parents of children with special educational needs (SEN) or psychiatric diagnoses, who frequently experience clinically significant distress and social isolation [4,5,6]. Negative outcomes have been documented among families of individuals with schizophrenia [7], as well as the stigma associated with mental illness among family caregivers [8]. A central yet often overlooked factor within family dynamics is co-dependency, characterized by maladaptive patterns of emotional suppression, interpersonal control, and excessive self-sacrifice [9,10,11,12,13]. Although the links between parental stress, self-efficacy, and well-being are well established [14,15,16], research has yet to clarify how these factors interact within diverse developmental contexts.

Concerning parents of children with special educational needs (SEN), substantial evidence indicates compromised parental well-being. Disabilities not only intensify emotional distress but also reduce coping capacity and access to social support [16]. These outcomes are closely related to the child’s condition, parental coping strategies, and the availability of social and professional support [5]. Among these parents, the risk of mental disorders, marital dissolution, job loss, and deteriorated mental health is significantly increased [17]. Parents of children with Down syndrome, genetic disorders, cerebral palsy, Rett syndrome, and intellectual disabilities also face additional emotional challenges, financial strain, and social isolation [16,18,19,20]. More broadly, parents of children with intellectual disabilities, including Down syndrome, experience higher levels of stress compared to parents of typically developing children, particularly when they are unemployed [21]. Parents of children with Down syndrome frequently report feelings of shame and guilt [16,22].

Parents of children with autism spectrum disorder (ASD) are reported to experience lower quality of life compared to parents of typically developing children [23,24,25] and are at increased risk of depression [26]. They also exhibit higher levels of stress, not only relative to parents of typically developing children but also compared to parents of children with other developmental disorders, consistently reporting heightened anxiety, stress, and need for support [16,27,28,29,30]. The demands associated with ASD caregiving appear to affect mothers more strongly [31]. In view of these strains, the importance of active coping strategies and functional social support is increasingly emphasized [32].

Addictions also affect the entire family system and generate relationship strain, psychological distress, and financial difficulties, as well as feelings of guilt, shame, and stigma among family members, underscoring the need for family-oriented interventions [33,34,35]. Research on stigma and co-dependency highlights caregiver stress, perceived stigma, and compromised mental health among female family members of individuals with addiction [36,37]. Co-dependency has been most extensively studied in families of individuals with substance use and alcohol addiction [38], particularly among mothers and wives with lower educational attainment or unemployment [33].

Family functioning, personality type, and co-dependency are conceptualized as interrelated constructs [39,40], and the concept of co-dependency has evolved beyond its origins in addiction research [9,12,13]. It is now understood as a psychological, medical, educational, and social issue involving learned behaviors and beliefs that hinder adaptive functioning [11]. Parents, children, and caregivers may all be vulnerable to various forms of co-dependent behavior [12], and increased parental self-efficacy can mitigate problematic behaviors [41]. Co-dependency is documented among caregivers of individuals with mental disorders [42] and is recognized as an unhealthy relational pattern [43,44] that negatively affects the quality of life of family members, particularly children [44,45]. Individuals with higher levels of co-dependency report more problematic relationships [46], whereas lower co-dependency is associated with greater relationship satisfaction [44,47]. Behavioral problems in childhood may escalate when family dysfunction is present [44,48], and systemic theories emphasize that children’s behavioral difficulties may signal dysfunction within the marital subsystem [44,49]. Related constructs such as family symbiosis [11] and parentification [50] further illustrate how dysfunctional relational patterns shape long-term psychological outcomes. Parental stress and well-being predict lower self-efficacy [15,16]; thus, parental resources constitute an essential prerequisite for supporting children and their development [51,52,53,54].

The aim of this study was to provide a person-centered understanding of parental psychological functioning across four family contexts: parents of children with special educational needs (SEN), parents of addicted children, parents of children with ASD and/or another psychiatric disorder, and parents of typically developing children. The study incorporated a comprehensive set of functional indicators (locus of control, well-being, satisfaction with life, flourishing, self-efficacy, mindfulness, self-esteem), dysfunctional indicators (general and child-specific co-dependency, fear of happiness, loneliness, avoidance, self-victimization, hopelessness), and sociodemographic characteristics (education, work status, family status, living situation, gender).

Although substantial research has documented the psychological strain experienced by parents across specific clinical groups, existing findings remain fragmented, typically focusing on isolated constructs or single diagnostic categories. A multidimensional, person-centered approach is therefore needed to capture how functional and dysfunctional psychological dimensions co-occur within parents and whether these configurations differ across diverse family contexts. Such an approach also enables the examination of how sociodemographic factors shape parents’ likelihood of belonging to particular psychological profiles.

Guided by this rationale, the present study addresses the following research questions:

- RQ1—Patterns of resourceful functioning—What patterns of resourceful functioning characterize parents across clinical and typical developmental contexts?
- RQ2—Group differences—How do parents of children with psychiatric diagnoses, parents of children with special educational needs, parents of addicted children, and parents of typically developing children differ in their psychological functioning profiles?
- RQ3—Sociodemographic predictors—Which sociodemographic factors predict membership in each psychological functioning profile?

## 2. Materials and Methods

### 2.1. Sample

The study was conducted online between February and June 2025 using the Survs platform (https://survs.com/). All participants provided digital informed consent prior to their engagement with the study, in accordance with the approved ethical protocol. Parents of children with addiction and those of children with special educational needs were recruited through the centers providing services to their children, while parents of children with ASD or other psychiatric conditions were recruited through a university psychiatric clinic. Parents of typically developing children were approached via schoolteachers. A total of 1000 invitations were distributed, yielding 310 responses, of which 281 contained complete data. The sample consisted predominantly of women (N = 248, 89.9%), with men representing 10.1% (N = 28). Most participants had higher education (N = 182, 67.2%), followed by secondary education (N = 87, 32.1%) and a very small proportion with primary education (N = 2, 0.7%). In terms of family status, 51.8% were married (N = 141), 32.4% cohabiting (N = 88), 6.6% separated (N = 18), 4.8% lone parents (N = 13), and 4.4% single (N = 12). Regarding living arrangements, 61.2% lived with other family members (N = 150), while 38.8% lived alone with their child (N = 95). The parental groups included parents of typically developing children (N = 146, 52.0%), parents of children with psychiatric disorders (N = 82, 29.2%), parents of children with SEN (N = 29, 10.3%), and parents of children with addictions (N = 24, 8.5%). Regarding employment status, most participants were employed (N = 197, 73%), while 9% were simultaneously studying and working (N = 24). A smaller proportion were not employed (N = 20, 7.4%), and 10% were neither studying nor working (N = 27). For all clinical subgroups (ASD, SEN, psychiatric diagnoses, and addiction), inclusion required that the child’s diagnosis had been formally established for at least one year prior to participation. The ASD group consisted exclusively of parents whose children had a confirmed ASD diagnosis, independent of any co-occurring psychiatric conditions. Within the clinical subgroup of 82 diagnostic records, a clear predominance of autism spectrum disorder (ASD) was observed, with code 6A02 accounting for 53.66% (*n* = 44) of the cases. Other significant categories included mixed developmental disorders (6A03; 13.41%, *n* = 11) and specific developmental disorder of motor function (6A00.1; 10.98%, *n* = 9). Less common diagnoses comprised mild intellectual disability without behavioral complications (6A00.0; 7.32%, *n* = 6), specific learning disorders (6A03.3; 6.10%, *n* = 5), and attention-deficit/hyperactivity disorder (6A05; 4.88%, *n* = 4). Rare diagnoses, including 6B20, 6A00.2, and 6A00, represented isolated cases (approximately 1.22% each), ensuring that the cumulative total aligns accurately with the defined sample of 82 parents of children with psychiatric disorders and maintains a strict distinction from the separate category of parents of children with special educational needs (SEN) and parents of addicted children, who had no psychiatric diagnosis. Parents in the typical development subgroup were recruited via schoolteachers, who identified and invited participants based on the inclusion criterion that no developmental challenges or restrictions had been reported or observed for their children.

### 2.2. Instruments

Administered scales included established and newly developed scales (Table 1). Established scales were used to measure composite co-dependency, loneliness, mindfulness, self-efficacy, fear of happiness, self-esteem, hopelessness, life orientations (life of pleasure and life of meaning), life satisfaction, and flourishing. Newly developed scales include child-specific co-dependency, avoidance, and self-victimization. To assess co-dependency specifically within the parent–child relationship, the Child-specific Co-dependency Scale was used, comprising two subscales: Self-Sacrifice for Child, measuring parental prioritization of the child’s needs, and Responsibility, capturing perceived parental obligation for the child’s emotions, behavior, and decisions. Avoidant coping was measured with the Avoidance Coping Scale, developed to assess cognitive and behavioral avoidance strategies. Perceived self-victimization was measured using the Self-Victimization Scale, comprising three subscales: General Distrust, Underestimation by Others, and Unexpected External Troubles. In addition, single-item measures were included: general perceived happiness and assessment of perceived happiness compared to peers; and three dimensions of locus of control—focused on family, external and internal focused—were included. All instruments employed Likert-type response formats ranging from “strongly disagree” to “strongly agree”. In this sample, the Rosenberg Self-Esteem Scale yielded a two-factor structure (positive and negative self-esteem), which were treated as separate indicators in subsequent analyses.

The psychometric results indicate generally acceptable reliability and structural validity for most instruments. Despite the statistical limitations observed in the absolute fit of a few short scales, all measures were retained given their high internal consistency and the person-centered nature of the planned Latent Profile Analysis.

### 2.3. Data Processing

Data processing and analyses were conducted using Jamovi version 2.6.44. All instruments—both existing and newly constructed—underwent Exploratory Factor Analysis (EFA), Confirmatory Factor Analysis (CFA), and reliability assessment using Cronbach’s alpha (α) and McDonald’s omega (ω). Following scale validation, descriptive statistics were computed for all variables to summarize central tendencies and dispersion. Distributions were examined to identify potential extreme values. As part of the preliminary analyses, paired comparisons and partial correlations were conducted to examine the distinctiveness and interrelations among conceptually related psychological constructs. Missing data were handled using pairwise deletion for descriptive statistics. To identify distinct psychological subgroups within the sample, Latent Profile Analysis was conducted on the full available sample (N = 281) using all cases with complete data on the profile indicators. This involved testing different numbers of latent profiles, specifically examining solutions with two, three, four, and five classes. Model selection was guided by multiple fit indices, including the Akaike Information Criterion (AIC), Bayesian Information Criterion (BIC), and the Bootstrap Likelihood Ratio Test (BLRT), alongside considerations of entropy and theoretical interpretability. Analysis of Variance (ANOVA) and Welch’s F-test were employed to assess significant differences in scale means across the latent classes, with Welch’s F-test used when assumptions of homogeneity of variances were violated. Finally, to explore the factors influencing latent class membership, regression analyses were conducted. These analyses incorporated demographic variables as predictors.

## 3. Results

### 3.1. Descriptive Statistics

Descriptive statistics for all psychological variables are presented in Table 2. Sample sizes ranged from 240 to 281 due to minimal missing data handled via pairwise deletion.

### 3.2. Latent Profile Analysis and Profile Characteristics

Latent Profile Analysis was conducted to identify distinct psychological configurations. Fit indices for the 2-, 3-, 4- and 5-profile solutions are presented in Table 3. The two-class model produced two broad groups—71% low functioning and low well-being, high co-dependency and low resource functioning; and 29% high well-being, low co-dependency and high resource functioning, and showed good classification accuracy (Entropy = 0.936). However, it offered only a distinction between low and high optimal functioning. The three-class model demonstrated improved fit (BIC = 8634; SABIC = 8311; Entropy = 0.901) and yielded a more differentiated and psychologically meaningful structure, consisting of the low well-being, high co-dependency and low resource functioning group (50%); the high well-being, low co-dependency and high resource functioning group (26%); and the moderate well-being, moderate co-dependency and moderately resourceful functioning group (24%). Although the four- and five-class models showed lower AIC and BIC values, both solutions produced fragmented and internally inconsistent profiles. In the five-class model, for example, Class 2 and Class 5 showed the highest flourishing scores, Class 3 showed the highest internal locus of control and life satisfaction, and Class 5 showed the highest life-of-pleasure scores, indicating a lack of coherent cross-indicator structure. Similarly, the four-class solution yielded classes that were “highest” on different indicators without forming stable or interpretable psychological patterns. These inconsistencies suggest statistical over-extraction rather than meaningful subgroup differentiation. The three-class model provided the most coherent, interpretable, and psychologically robust solution.

**Figure 1:**
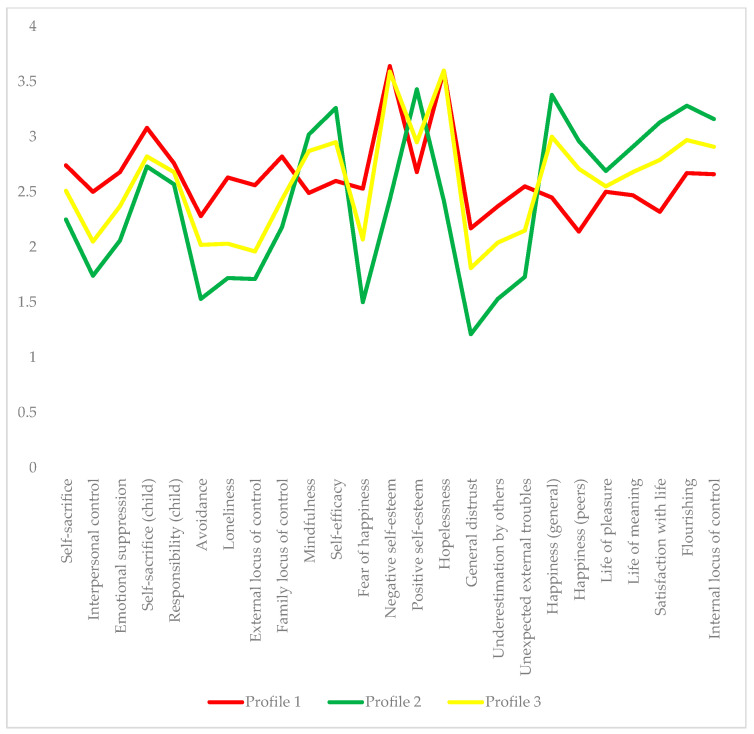
Profiles of parents.

Figure 1 illustrates the three-profile solution and profiles of the parents.

Mean comparisons across the three latent profiles revealed consistent and theoretically meaningful distinctions in psychological functioning. Profile 1 exhibits the highest psychological vulnerability. Profile 2 represents a high well-being, low co-dependency and high resource functioning and emotionally open profile; and Profile 3 takes an intermediate or moderate well-being, moderate co-dependency and moderately resourceful functioning position (Table 4).

Profile 1: Low well-being, high co-dependency and low resource functioning (50%): This profile showed the most dysfunctional pattern, described by highest composite co-dependency measures (self-sacrifice, interpersonal control, and emotional suppression); child-specific co-dependency (self-sacrifice and responsibility); avoidance, loneliness, fear of happiness, low self-esteem, and self-victimization measures (general distrust, underestimation by others, unexpected external troubles); and family and external locus of control; and lowest self-esteem, mindfulness, self-efficacy, happiness, life of pleasure, life of meaning, satisfaction with life, flourishing and internal locus of control. Hopelessness is higher compared to Profile 2 and the same as Profile 3.

Profile 2: High well-being, low co-dependency and high resource functioning (26%): This group demonstrated the most functional configuration, with lowest composite co-dependency measures (self-sacrifice, interpersonal control, and emotional suppression); child-specific co-dependency (self-sacrifice and responsibility); avoidance, loneliness, fear of happiness, low self-esteem, and self-victimization measures (general distrust, underestimation by others, unexpected external troubles); hopelessness; and family and external locus of control; and highest self-esteem, mindfulness, self-efficacy, happiness, life of pleasure, life of meaning, satisfaction with life, flourishing and internal locus of control.

Profile 3: Moderate well-being, moderate co-dependency and moderately resourceful functioning (24%): This group occupied an intermediate position with moderate levels of composite co-dependency measures (self-sacrifice, interpersonal control, and emotional suppression); child-specific co-dependency (self-sacrifice and responsibility); avoidance, loneliness, fear of happiness, low self-esteem, and self-victimization measures (general distrust, underestimation by others, unexpected external troubles); and family and external locus of control, self-esteem, mindfulness, self-efficacy, happiness, life of pleasure, life of meaning, satisfaction with life, flourishing and internal locus of control. Hopelessness is lower compared to Profile 2 and the same as Profile 1.

### 3.3. Between-Profile Differences and Profile Membership

Welch’s ANOVA indicated significant mean differences across the three latent profiles for four key variables: happiness compared to peers, general distrust, hopelessness, and low self-esteem (Table 5). Profile 2 (High well-being, low co-dependency and high resource functioning) showed the most adaptive pattern, with significantly lower levels of general distrust, hopelessness, and low self-esteem, as well as higher happiness compared to peers relative to both Profile 1 (Low well-being, high co-dependency and low resource functioning) and Profile 3 (Moderate well-being, moderate co-dependency and moderately resourceful functioning). Profile 1 demonstrated the highest levels of psychological distress, showing the lowest happiness compared to peers and the highest general distrust, hopelessness, and low self-esteem. Profile 3 occupied an intermediate position overall, although it matched Profile 1 on hopelessness and low self-esteem, while showing lower general distrust and higher happiness compared to peers. Marginal trends were observed for fear of happiness, underestimation by others, and unexpected external troubles. Standardized residual analysis further showed that individuals from the clinical subgroups were overrepresented in the low well-being, high co-dependency and low resource functioning profile, supporting the validity of the identified classes. The absence of significant univariate differences for most indicators is consistent with the logic of Latent Profile Analysis. LPA identifies subgroups based on the multivariate pattern of responses rather than on isolated mean differences for each variable. Thus, even when individual indicators do not differ significantly on their own, their combined configuration forms distinct and theoretically meaningful profiles.

A multinomial logistic regression model was statistically significant, χ^2^(36) = 62.7, *p* = 0.004, explaining a moderate proportion of variance (McFadden R^2^ = 0.134) (Table 6). Predictors with extremely sparse categories (*n* = 2–5) were removed due to quasi-complete separation and estimation instability.

When parental background was examined as a predictor of profile membership, parents of children with psychiatric diagnoses served as the reference group in all comparisons. Relative to this group, parents of children with addiction showed a non-significant trend toward being less likely to belong to the Low Well-Being, High Co-Dependency, and Low Resource Functioning profile than to the High Well-Being, Low Co-Dependency, and High Resource Functioning profile. Although the odds ratio suggested a tendency toward the high-functioning profile, the confidence interval included 1, indicating that this effect should be interpreted cautiously. In contrast, individuals who were neither studying nor working were significantly more likely to fall into the most distressed profile rather than the high-functioning profile.

When comparing the Moderate Well-Being, Moderate Co-Dependency, and Moderately Resourceful Functioning profile to the high-functioning profile (with Profile 2 as the reference), parents of typically developing children were substantially more likely to belong to the moderate-functioning group than parents of children with psychiatric diagnoses. This pattern was even stronger when comparing the moderate-functioning profile to the most distressed profile (with Profile 1 as the reference): parents of typically developing children were more likely to fall into the moderate profile than into the low well-being profile. Conversely, individuals who were not studying and not working were significantly less likely to be part of the moderate-functioning profile than the most distressed one, suggesting that lack of occupational engagement is specifically associated with the lowest-functioning group. Finally, separated parents showed a marginal, non-significant tendency to be more likely to belong to the moderate-functioning profile rather than the low well-being profile, relative to married parents.

## 4. Discussion

The present study addressed three research questions. First, we examined the characteristic patterns of high and low resourceful functioning among parents across clinical and typical developmental contexts (RQ1). Second, we investigated whether parents of children with autism or psychiatric diagnoses, parents of children with special educational needs, parents of dependent adolescents, and parents of typically developing children differ in their likelihood of belonging to these profiles (RQ2). Third, we explored which parental circumstances and sociodemographic factors predict membership in each profile (RQ3).

Regarding RQ1: The Latent Profile Analysis identified three profiles—low well-being, high co-dependency and low resource functioning; high well-being, low co-dependency and high resource functioning; and moderate well-being, moderate co-dependency and moderately resourceful functioning—highlighting substantial heterogeneity in parental psychological functioning that extends beyond traditional diagnostic categories. Profile 1: Low well-being, high co-dependency and low resource functioning (50%). The first profile reflected the most maladaptive psychological configuration in the sample. Individuals in this group showed a pronounced pattern of co-dependency, expressed through elevated tendencies toward self-sacrifice, interpersonal control, and emotional inhibition, as well as heightened dependence on the child. Their emotional and behavioral functioning was further characterized by avoidance, loneliness, fear of happiness, and low self-esteem. They also displayed stronger self-victimization tendencies, including distrust, feelings of being underestimated, and expectations of adverse external events, accompanied by a predominantly external and family-focused locus of control. In contrast to these vulnerabilities, resources were diminished. Participants in this profile reported the lowest levels of mindfulness, self-efficacy, happiness, meaning and pleasure in life, life satisfaction, flourishing, and internal locus of control. Hopelessness was elevated relative to the high-functioning profile and comparable to that observed in the moderately functioning group. Profile 2: High well-being, low co-dependency and high resource functioning (26%). This profile reflected the most adaptive psychological configuration observed in the study. Members of this group consistently reported the lowest levels of composite co-dependency, including tendencies toward self-sacrifice, interpersonal control, and emotional suppression, as well as reduced child-focused co-dependency in the form of self-sacrifice and responsibility. They also showed minimal avoidance, loneliness, fear of happiness, and low self-esteem, together with lower endorsement of self-victimization indicators such as general distrust, feelings of being underestimated, and expectations of external troubles. Hopelessness and reliance on family or external locus of control were likewise diminished. In contrast to these vulnerabilities, protective psychological resources were most strongly expressed in this profile. Members demonstrated the highest levels of self-esteem, mindfulness, and self-efficacy, alongside greater happiness, pleasure, and meaning in life. Satisfaction with life and flourishing were also most pronounced, accompanied by a predominant internal locus of control. Taken together, this constellation of low-risk and high-protective indicators underscores the resilience and functional capacity of this group, positioning it as the most well-functioning profile within the sample. Profile 3: Moderate well-being, moderate co-dependency and moderately resourceful functioning (24%). This profile occupied an intermediate position within the overall configuration of parental functioning. Members of this group reported moderate levels of positive indicators such as mindfulness, self-efficacy, self-esteem and flourishing, yet their scores on negative indicators remained elevated. Hopelessness resembled the pattern observed in the Low Well-Being, High Co-Dependency and Low Resource Functioning profile. At the same time, distrust and avoidance were somewhat lower than in the most distressed group, though well-being indicators were consistently weaker than those found in the High Well-Being, Low Co-Dependency and High Resource Functioning profile. This mixed pattern suggests a form of emotional ambivalence: partial coping resources are present, but they coexist with persistent self-doubt and vulnerability. The profile reflects a state of psychological inconsistency, where resources are insufficient to offset accumulated strain. These findings are consistent with research emphasizing the multifaceted nature of parental stress and well-being [2,3,5]. The identification of the low well-being, high co-dependency and low resource functioning profile, characterized by high levels of emotional suppression and interpersonal control, is particularly important given its high prevalence. According to Christou and Bacopoulou [1], the emotional state of the parent is not merely a psychological backdrop but a physiological regulator within the relational system. When parents chronically suppress their emotions—as observed in our most vulnerable subgroup—they may inadvertently compromise the neurocognitive pathways that facilitate co-regulation and attachment security. This suggests that the parental pattern identified in this study should inform future research and the development of family-centered interventions that target parental self-regulation as a prerequisite for child development.

Concerning RQ2: In examining predictors of profile membership, several clear contrasts emerged when parental background, work status, and family situation were considered relative to parents of children with psychiatric diagnoses, who served as the reference group in all comparisons. Parents of children with addiction showed a non-significant trend toward being less likely to belong to the Low Well-Being, High Co-Dependency and Low Resource Functioning profile than to the High Well-Being, Low Co-Dependency and High Resource Functioning profile, indicating a tendency to align more often with the high-functioning group. By contrast, parents of typically developing children showed a strong tendency to cluster in the Moderate Well-Being, Moderate Co-Dependency and Moderately Resourceful Functioning profile: they were over ten times more likely to be in Profile 3 than in Profile 2, and nearly thirty times more likely to be in Profile 3 than in Profile 1, again relative to parents of children with psychiatric diagnoses. This pattern underscores that parents of children with psychiatric diagnoses are comparatively underrepresented in the moderate profile and more often situated at the extremes of functioning. The low well-being, high co-dependency and low resource functioning profile aligns with research reporting elevated distress among parents of children with ASD, SEN, and chronic conditions, who frequently report heightened anxiety, depression, and social isolation [24,29]. Its emphasis on emotional suppression, interpersonal control, and self-sacrifice is consistent with transdiagnostic models of co-dependency [9,12,13]. The finding that parents of children with ASD were significantly more likely to belong to the Low Well-Being, High Co-Dependency and Low Resource Functioning profile—marked by high hopelessness and low self-esteem—aligns with existing evidence on the role of self-stigma [65] and internalized stigma, which are identified as major sources of strain for primary caregivers [25]. Furthermore, given the female-dominant nature of our sample (89.9%), these results underscore a specific maternal vulnerability. Mothers of autistic children frequently report higher rates of depression than the general population [66], with maternal self-stigma identified as a critical contributor to these depressive symptoms [67]. This aligns with research identifying hopelessness and self-critical tendencies as central markers of parental distress [18,19,31], and with evidence that distrust and social isolation are core features of vulnerable family systems [6,35]. Thus, the identified low well-being, high co-dependency and low resource functioning profile may reflect the cumulative impact of chronic caregiving stress and societal stigma on maternal psychological well-being. The high well-being, low co-dependency and high resource functioning profile aligns with literature on the protective role of parental self-efficacy, social support, and positive affect [15,16]. Happiness compared to peers distinguished the high well-being, low co-dependency and high resource functioning and moderate well-being, moderate co-dependency and moderately resourceful functioning profiles from the low well-being, high co-dependency and low resource functioning group, consistent with literature showing that strong peer networks buffer against maladaptive coping and co-dependency [15,51]. Membership of parents of typically developing children in the Moderate profile is consistent with research stressing immature defenses in non-clinical contexts and suggesting that co-dependency in non-clinical contexts may have more favorable characteristics [13,68].

With respect to RQ3: Work engagement also differentiated the profiles. Individuals who were neither studying nor working were almost four times more likely to belong to Profile 1 than Profile 2, and less likely to be in Profile 3 than in Profile 1. This pattern suggests that lack of occupational activity is specifically associated with the most dysfunctional profile and reduces the likelihood of moderate functioning, aligning with evidence that socioeconomic inactivity is linked to heightened psychological vulnerability and reduced access to coping resources [2,5].

Education was initially included as a predictor, in line with prior research showing that lower educational attainment is associated with increased parental stress and reduced access to supportive networks [16,24]. However, in this sample, the distribution of educational categories was too uneven to allow stable estimation, and the variable was therefore excluded from the final model.

Family status further contributed to profile differentiation. Separated parents showed a marginal tendency to be more likely in Profile 3 than in Profile 1, relative to married parents, pointing to a pattern where separation is linked to moderate functioning rather than severe dysfunction and supporting the outcomes outlined in the literature [69].

## 5. Limitations and Future Research

This study has several limitations. Its cross-sectional design prevents causal inference and does not allow examination of how parental psychological profiles change over time. Reliance on self-report measures introduces potential biases, and future work would benefit from longitudinal and qualitative approaches. The sample was strongly female-dominant (89.9%), reflecting broader caregiving patterns but limiting generalizability to fathers.

Although the LPA yielded three robust multivariate profiles, only four indicators showed significant univariate differences, meaning that the remaining variables function primarily as descriptive components of the broader multivariate configuration. Finally, three of the psychological scales (CAMS-R, Fear of Happiness, Hopelessness) showed poor CFA fit, which is common in short scales with few degrees of freedom. Because all three demonstrated high internal reliability, and LPA operates on observed indicators rather than latent structures, they were retained. Nonetheless, their limited structural fit represents a psychometric constraint that future research should address. Additionally, the interpretation of the latent profiles should be viewed as descriptive rather than diagnostic. Profile labels reflect relative patterns within this sample and may not generalize to other populations. Furthermore, the unequal distribution of parental subgroups (e.g., psychiatric, autistic, SEN, and typically developing) may have influenced profile membership probabilities and limits the generalizability of the findings.

## 6. Conclusions

The present study identified three distinct parental psychological profiles—high well-being, low co-dependency and high resource functioning; low well-being, high co-dependency and low resource functioning; and moderate well-being, moderate co-dependency and moderately resourceful functioning—revealing substantial heterogeneity in how parents navigate emotional, relational, and coping demands across diverse developmental contexts. Parents of children with ASD or psychiatric diagnoses emerged as the most vulnerable group, showing the highest likelihood of belonging to the low well-being, high co-dependency and low resource functioning profile, which underscores the need to shift from child-centered approaches toward family-centered models of support. At the same time, a considerable proportion of parents of typically developing children also exhibited low well-being, high co-dependency and low resource functioning or moderate well-being, moderate co-dependency and moderately resourceful functioning, indicating that contemporary parenting pressures extend psychological strain far beyond clinical contexts. These findings highlight that supporting the parent is a prerequisite for supporting the child, and that effective intervention must strengthen the emotional and relational resources of the entire family system.

A central finding of this study is the elevated psychological vulnerability, affecting 50% of the total sample. This high prevalence indicates that psychological strain is a parenting challenge, where even parents of typically developing children frequently exhibit suboptimal functioning regardless of the absence of a clinical diagnosis. Consequently, these results highlight that parental distress is a common phenomenon, highlighting the need for broader family-centered support approaches. By integrating psychological markers, sociodemographic factors, and parental contexts, this study offers a person-centered framework for understanding where parental well-being is most compromised and where targeted support is most urgently needed. Importantly, the identified profiles should be interpreted as descriptive rather than diagnostic categories, reflecting patterns specific to this sample. The results open a clear implication for expanding and deepening family-centered practices, with particular attention to the most vulnerable situations—especially families raising children with ASD or psychiatric diagnoses—while also recognizing that many parents, regardless of diagnostic context, may require structured emotional and relational support. Future research should build on these preliminary insights through longitudinal and multi-method designs, enabling a more precise understanding of how parental functioning evolves and how coordinated, systemic interventions can best promote resilience within families.

## Figures and Tables

**Table 1:** Psychometric properties and summary of study scales.

Scale Name	Included Items	Status/Source	Reliability (*α*/*ω*)	Key CFA Fit Indices
Composite Co-dependency Scale (CCS)	10	Established measure [10]	0.625–0.756	CFI = 0.960; TLI = 0.946; RMSEA = 0.050
Child-specific Co-dependency Scale	9	Newly developed for this study	0.695–0.748	CFI = 0.926; TLI = 0.898; RMSEA = 0.075
Avoidance Coping Scale	5	Newly developed for this study	0.827/0.831	CFI = N/A; TLI = 1.02; RMSEA = 0.000
3-Item UCLA Loneliness Scale	3	Established [55]	0.827/0.835	CFI = 1.00; TLI = 1.00; RMSEA = 0.000
Mindfulness (CAMS-R)	9	Established [56]	0.831/0.835	CFI = 0.830; TLI = 0.773; RMSEA = 0.131
Generalized Self-Efficacy Scale	3	Established [57,58]	0.798/0.799	CFI = 1.00; RMSEA = 0.000
Fear of Happiness Scale	4	Established [59]	0.862/0.865	CFI = 0.954; TLI = 0.862; RMSEA = 0.210
Self-Esteem Scale	6	Established [60]	0.765–0.826	CFI = 0.929; TLI = 0.896; RMSEA = 0.079
Beck Hopelessness Scale	4	Established [61]	0.844/0.855	CFI = 0.968; TLI = 0.903; RMSEA = 0.183
Self-Victimization Scale	16	Newly developed for this study	0.750–0.902	CFI = 0.946–0.972; RMSEA = 0.110–0.130
Orientations to Happiness	11	Established [62]	0.682–0.702	CFI = 0.869; TLI = 0.839; RMSEA = 0.063
Satisfaction with Life Scale	5	Established [63]	0.845/0.849	CFI = 0.981; TLI = 0.963; RMSEA = 0.085
Flourishing Scale	8	Established [64]	0.868/0.871	CFI = 0.923; TLI = 0.892; RMSEA = 0.110

**Table 2:** Descriptive statistics for psychological variables.

Variable	N	Mean	*SD*	Min	Max	Skewness	Kurtosis
Co-dependency							
Self-Sacrifice	281	2.52	0.597	1.00	4.00	0.088	0.063
Interpersonal control	280	2.10	0.618	1.00	4.00	0.496	0.548
Emotional suppression	279	2.37	0.584	1.00	4.00	0.040	0.193
Child-specific co-dependency							
Self-sacrifice child	278	2.83	0.560	1.00	4.00	−0.057	0.159
Responsibility child	281	2.65	0.505	1.00	4.00	0.265	1.090
Avoidance	278	1.97	0.568	1.00	4.00	0.265	0.224
Loneliness	277	2.16	0.659	1.00	4.00	0.252	0.187
Fear of happiness	279	2.07	0.655	1.00	4.00	0.064	−0.303
Low self-esteem	279	2.94	0.552	1.00	4.00	−0.543	1.540
Hopelessness	279	3.30	0.715	2.00	5.00	0.057	−0.475
Self-victimization							
General distrust	276	1.79	0.509	1.00	3.43	−0.046	−0.333
Underestimation by others	279	2.00	0.504	1.00	3.50	−0.130	0.037
Unexpected external troubles	277	2.15	0.547	1.00	3.50	−0.268	0.092
High self-esteem	279	3.33	0.762	2.00	5.00	−0.026	−0.611
Mindfulness	278	2.81	0.427	1.00	4.00	−0.479	2.480
Self-efficacy	279	2.88	0.512	1.00	4.00	−0.081	0.296
Happiness (general)	276	2.92	0.667	1.00	4.00	−0.280	0.204
Happiness (peers)	276	2.66	0.719	1.00	4.00	0.209	−0.491
Life of pleasure	265	2.58	0.416	1.00	4.00	0.268	1.460
Life of meaning	266	2.69	0.445	1.20	4.00	0.363	1.000
Satisfaction with life	265	2.77	0.539	1.00	4.00	−0.145	1.120
Flourishing	240	2.96	0.417	1.38	4.00	−0.234	2.170
Internal Locus of Control	279	2.88	0.580	1.00	4.00	−0.210	−0.450
External Locus of Control	279	2.13	0.715	1.00	4.00	0.335	0.091
Family-focused Locus of Control	278	2.48	0.729	1.00	4.00	−0.065	−0.278

**Table 3:** Fit indices for the 2-, 3-, 4- and 5-class solutions.

Model	Classes	LogLik	AIC	BIC	SABIC	Entropy	n_min	n_max	ICL
2-class	2	−4187	8526	8789	8548	0.936	0.294	0.706	−8798
3-class	3	−4038	8281	8634	8311	0.901	0.238	0.502	−8658
4-class	4	−3922	8100	8543	8137	0.928	0.115	0.404	−8563
5-class	5	−3811	7930	8463	7975	0.907	0.089	0.264	−8496

**Table 4:** Mean estimates for the three latent profiles.

Variable	Class 1	Class 2	Class 3
Co-dependency			
Self-sacrifice	2.74	2.25	2.51
Interpersonal control	2.50	1.74	2.05
Emotional suppression	2.68	2.06	2.37
Child-specific co-dependency			
Self-sacrifice	3.08	2.73	2.82
Responsibility	2.76	2.57	2.68
Avoidance	2.28	1.53	2.02
Loneliness	2.63	1.72	2.03
Fear of happiness	2.53	1.50	2.07
Low self-esteem	3.64	2.43	3.59
Hopelessness	3.60	2.42	3.60
Self-victimization			
General distrust	2.17	1.21	1.81
Underestimation by others	2.37	1.53	2.04
Unexpected external troubles	2.55	1.73	2.15
High self-esteem	2.68	3.43	2.95
Mindfulness	2.49	3.02	2.87
Self-efficacy	2.60	3.26	2.95
Happiness (general)	2.45	3.38	3.00
Happiness (peers)	2.14	2.96	2.71
Life of pleasure	2.50	2.69	2.55
Life of meaning	2.47	2.91	2.68
Satisfaction with life	2.32	3.13	2.79
Flourishing	2.67	3.28	2.97
Internal locus of control	2.66	3.16	2.91
External locus of control	2.56	1.71	1.96
Family locus of control	2.82	2.18	2.43

**Table 5:** Significant differences among psychological profiles.

Variable	Class Means (C1, C2, C3)	Significant Pairwise Differences (Games-Howell)
Happiness Compared to Peers	C1: 2.14; C2: 2.96; C3: 2.71	C2 > C1 (*p* < 0.001); C3 > C1 (*p* < 0.001)
General Distrust	C1: 2.17; C2: 1.21; C3: 1.81	C2 < C1 (*p* < 0.001); C2 < C3 (*p* < 0.001); C3 < C1 (*p* < 0.001)
Hopelessness	C1: 3.60; C2: 2.42; C3: 3.60	C2 < C1 (*p* < 0.001); C2 < C3 (*p* < 0.001)
Low self-esteem	C1: 3.64; C2: 2.43; C3: 3.59	C2 < C1 (*p* < 0.001); C2 < C3 (*p* < 0.001)

Note: C1 = Low well-being, high co-dependency and low resource functioning; C2 = High well-being, low co-dependency and high resource functioning; C3 = Moderate well-being, moderate co-dependency and moderately resourceful functioning.

**Table 6:** Multinomial logistic regression.

Predictor (Category vs. Reference)	Estimate	*p*-Value	Odds Ratio	95% CI Lower	95% CI Upper
Reference category: Profile 2. Odds Ratio (OR) > 1 indicates a higher likelihood of membership in Profile 1 relative to Profile 2; OR < 1 indicates a lower likelihood					
(Parental Background: vs. Parents of children with psychiatric diagnoses)					
Parents of children with addiction (vs. Parents of children with psychiatric diagnoses)	−1.7166	0.056	0.1797	0.0309	1.0433
Work Status					
Not Studying and Not Working (vs. Working)	1.3299	0.045	3.7806	1.0320	13.8503
Reference category: Profile 2. Odds Ratio (OR) > 1 indicates a higher likelihood of membership in Profile 3 relative to Profile 2; OR < 1 indicates a lower likelihood.					
(Parental Background: vs. Parents of children with psychiatric diagnoses)					
Parents of typically developing children (vs. Parents of children with psychiatric diagnoses)	2.3401	0.004	10.3823	2.0841	51.7209
Reference category: Profile 1. Odds Ratio (OR) > 1 indicates a higher likelihood of membership in Profile 3 relative to Profile 1; OR < 1 indicates a lower likelihood.					
(Parental Background: vs. Parents of children with psychiatric diagnoses)					
Parents of typically developing children (vs. Parents of children with psychiatric diagnoses)	3.3344	<0.001	28.0622	4.7215	166.7865
Work Status					
Not Studying and Not Working (vs. Working)	−1.6915	0.046	0.1842	0.0350	0.9690
Family Status					
Separated (vs. Married)	1.9596	0.061	7.0966	0.9169	54.9240

## Data Availability

Data supporting the findings of this study are available within the article. Further information can be requested from the corresponding authors. Due to privacy considerations, additional data are not publicly available.
